# Based on dual perspectives of management and ethics: exploring challenges and governance approaches for new media applications in psychiatric specialty hospitals

**DOI:** 10.3389/fdgth.2026.1729242

**Published:** 2026-05-04

**Authors:** Qiuqing Wen, Yingying Shen

**Affiliations:** 1 Medical Department, Huzhou Third Municipal Hospital, The Affiliated Hospital of Huzhou University, Zhejiang, China; 2 Information Technology Department, Huzhou Third Municipal Hospital, The Affiliated Hospital of Huzhou University, Zhejiang, China

**Keywords:** artificial intelligence (AI), doctor-patient relationship, new media, privacy ethics, psychiatric hospitals

## Abstract

The further promotion and application of new media technologies present new opportunities for psychiatric specialty hospitals in areas such as health education, doctor-patient communication, service extension, and brand building. However, due to the unique characteristics and sensitivity of the mental health field, the challenges faced by new media applications in this domain far exceed those encountered in general hospitals. Based on dual perspectives of management and ethics, this paper explores several core challenges in the new media practices of psychiatric hospitals, including: privacy risks and lack of ethical oversight, power shifts and responsibility ambiguity, difficulties in the effective dissemination of health information, and sustainability issues in operation under resource constraints. To address these challenges, this study proposes measures including: constructing a “privacy protection technology + layered informed consent management” mechanism, standardizing the boundaries of online consultation services, establishing an AI-assisted “hierarchical knowledge base” system for dissemination, and improving a “full-cycle ethical risk control system.” The focus lies in clarifying the essential nature of the problems faced by psychiatric hospitals. This paper emphasizes that, under the premise of adhering to ethical bottom lines, constructing a dedicated management system tailored to the mental health field is the essential path to achieving high-quality hospital development empowered by new media and effectively safeguarding patient rights and interests.

## 1 Introduction

With the continuous updating and iteration of information technology, the application of new media platforms in the healthcare field is becoming increasingly widespread. To actively implement the spirit of documents like the “Healthy China 2030” Plan Outline, psychiatric specialty hospitals are also attempting to use information technology to transform medical service models and expand the boundaries of health education and services. The “new media technology” mentioned in this paper is a dynamically evolving, interdisciplinary frontier field. It is not merely a tool but a core driver reshaping information dissemination, social interaction, and business models. It primarily refers to an emerging set of technologies based on the internet and digital technology, used for creating, disseminating, and interacting with digital content (such as text, images, audio, and video). This includes platforms and applications that enable two-way interaction, real-time communication, and user-generated content, such as official social media accounts of medical institutions, health education applications, and online consultation platforms. However, due to the high sensitivity of the mental health field and issues such as social discrimination, their new media applications face more complex challenges. Taking the ranking of new media accounts within the Zhejiang Provincial Health System as an example, from February 2023 to January 2024, only Hangzhou Seventh People's Hospital made the list three times among all levels of psychiatric specialty hospitals in the province, while other similar specialty hospitals were absent from the rankings. Beyond the difficulties in advancing health education, psychiatric hospitals face risks distinct from those in general hospitals concerning data privacy, patient compliance, and social discrimination. These risks indirectly indicate that the application of new media in psychiatric hospitals is not merely a simple technological transplant, but a transformation requiring optimized internal management and strengthened ethical protection.

## 2 Core challenges of new media application in psychiatric hospitals

New media plays an increasingly important role in disseminating health knowledge, enhancing patient experience, and improving public health levels. Its construction and operation have become a significant development direction for the healthcare industry (1). However, due to the specific nature of their professional field, psychiatric hospitals face unique risk challenges while using new media to improve medical services.

### 2.1 Privacy security and lack of ethical oversight

Hospital current HIS systems mostly use patient ID as the unique index, making the data highly sensitive. Once leaked, it can easily lead to employment discrimination and social exclusion, causing “secondary harm” to patients. Although various anonymization measures can be adopted for communication channels provided by diverse social platforms, it remains difficult to completely prevent the risk of information being identified through cross-referencing. Furthermore, once information is published, it is difficult to erase completely. This characteristic of “leaving traces” also creates a lasting psychological burden for user groups (2).

The original intention of algorithm recommendation is to provide precise push based on user profiles to enhance user satisfaction and stickiness (3). However, for patient groups in specific disease states, it can form ethical traps due to algorithmic models and data processing, potentially overexposing them to negative information environments and constituting a potential inducement risk (4).

Furthermore, ethical reviews in most hospitals are typically confined to traditional medical research. There is a lack of systematic supervision and ethical review (5) for potentially high-risk aspects of new media operations, including content publication, online interaction, and data reuse. Hospitals also generally lack effective internal monitoring mechanisms, leading to operational difficulties in management practices and severely restricting the compliance and risk control capabilities of new media applications.

### 2.2 Power shift and responsibility ambiguity

While new media applications change the existing doctor-patient relationship, they also bring risks of blurred medical responsibility and power shift. Although online consultation is convenient and private, its “de-contextualized” nature can easily lead to misdiagnosis or delayed intervention by doctors, increasing the risk of treatment failure for patients.

While internet hospitals improve the efficiency of doctor-patient communication, they also create an illusion of being “always online” and providing “instant responses” for patients. Some platforms, to enhance user engagement and satisfaction (6), have also set up SMS reminder functions, and even display the doctor's response time on the consultation interface for users to evaluate and choose. This invisible pressure can easily trigger professional burnout among medical staff, leading to decreased response quality and affecting the process of patients seeking medical advice.

### 2.3 Precision paradox and dissemination attenuation

Psychiatric hospitals use new media platforms for health science popularization aiming to change the public's biased views on mental illness and reduce social stigma. However, the dissemination process may unintentionally reinforce labels, and even trigger platform algorithms to strengthen covert discriminatory categorization of specific users, resulting in a precision paradox in dissemination outcomes.

Meanwhile, professional science content struggles to balance credibility and readability, and is easily drowned out by massive information during dissemination, making it difficult to effectively reach the truly needy target audience. This situation of “highly scarce attention resources” results in a low input-output ratio. How to carve out a unique position for the psychiatric specialty field within the fiercely competitive science popularization landscape becomes a management challenge now and in the future.

### 2.4 Resource constraints and operational challenges

Psychiatric hospitals also face limitations due to resource constraints in the process of new media operation. Producing a single piece of scientific, rigorous, vivid, and compliant high-quality science content requires significant investment of human and material resources, but the direct benefits generated from dissemination are very low, making it difficult to obtain sustained and stable support within the existing budget system of public hospitals. Furthermore, supporting high-quality new media operations often requires interdisciplinary talent. However, individuals proficient in psychiatry, communication studies, data security, and medical ethics are scarce (7), making it difficult to meet the comprehensive demands of high-quality content output and long-term stable platform operation. Coupled with rapid technological iteration, hospitals generally lack professional teams for strategy optimization, leading to inefficient and repetitive operations, making it difficult to effectively enhance efficiency and adapt to environmental changes (8).

## 3 Constructing a new media management system adapted to the mental health field

### 3.1 Technology empowerment and institutional innovation

To further strengthen privacy security defenses and rebuild the cornerstone of doctor-patient trust, considering the current level of domestic information technology development, psychiatric hospitals can consider adopting a dual-driven development strategy combining technology and institution:

1. Technically, attempt to develop or collaborate with third parties to introduce advanced privacy protection technologies, performing anonymization and desensitization processing on sensitive data to ensure maximum protection of patient information while utilizing data to improve services. For example, Federated Learning can be integrated with hospital HIS systems to establish a “central-node” collaborative architecture. The federated learning management platform serves as the coordinator, aggregator, and task manager, responsible for defining training tasks, distributing initial models and training protocols, securely aggregating encrypted model updates from nodes, and evaluating global performance while managing the entire workflow. Hospital nodes, deployed within the internal network, utilize federated learning clients to securely extract, desensitize, and align required data from systems such as HIS, PACS, LIS, and EMR through a medical information integration engine. Locally, these nodes employ technologies such as homomorphic encryption and differential privacy to train global models, generating encrypted model updates and exchanging them with the central platform via secure channels (9). However, it is important to note that while technologies like federated learning, homomorphic encryption, and differential privacy contribute to privacy protection, their application faces multiple challenges (10). At the regulatory and accountability level, the legal responsibility for harmful outputs from collaboratively developed federated models remains unclear, necessitating supporting laws and regulations for clarification. Technically, inconsistencies in data formats, quality, and annotation standards across hospitals may lead to model bias, affecting applicability to diverse patient groups and potentially contradicting ethical principles of fairness. In terms of sustainable operation, the high demands on computing resources, storage, and professional maintenance capabilities may exclude resource-constrained small and medium-sized specialized hospitals, exacerbating the “digital divide.” Therefore, while promoting the implementation of these technologies, efforts must simultaneously advance standardization, accountability clarification, and resource support systems. Additionally, the design and application of all AI-assisted systems must strictly comply with the baseline requirements of relevant domestic and international regulations, ensuring that technology genuinely serves patient well-being without compromising their dignity or autonomy.
  With the widespread application of AI in healthcare, traditional centralized training models face dual challenges of legal compliance and data security. “Federated learning” through its innovative architecture of “move the model to the data, not the data to the model” is reshaping the development path of medical AI. Therefore, collaborative learning technologies like federated learning can be deployed locally within hospitals, enabling model training without the need to aggregate original sensitive data. Applying homomorphic encryption technology allows necessary analytical computations to be performed on data while still encrypted, with only the final result decrypted, ensuring the original data remains protected throughout the process (9). Before publishing aggregated data, strictly controlled random perturbation methods such as differential privacy can be used, effectively preventing individual identification while ensuring the macro-usability of statistical results.
2. Institutionally, abandon the traditional “one-time checkmark consent” model. Design differentiated and adjustable authorization processes based on the sensitivity and potential risk level of data operations, using clear, intuitive, and non-technical language to explicitly explain the specific purpose, potential risks, and the user's rights (especially the right to withdraw consent at any time) for each data operation, ensuring that user authorizations are based on fully informed consent.

### 3.2 Standardizing online doctor-patient interaction mechanisms

In modern medical service models, medical institutions have long evolved from mere media “gatekeepers” to media “service providers” (11). Based on this shift in role positioning, to effectively manage the potential risks of online consultation for mental illnesses, hospitals should strictly control online inquiries according to the relevant requirements of the “Expert Consensus on Internet Diagnosis and Treatment in Psychiatry” (12), limiting the service scope to low-risk areas compliant with policy support, such as follow-up appointment scheduling, medication guidance, non-urgent psychological counseling, answers to recovery-phase questions, and health science information dissemination. Furthermore, it should be explicitly prohibited for hospitals to use new media platforms for initial diagnosis, complex condition diagnosis, suicide crisis intervention, adjustment of psychotropic medication prescriptions, and other high-risk behaviors, implementing checks at key medical links to prevent omissions. Simultaneously, to fully fulfill the duty of informed consent, hospitals should consistently display service scope limitations prominently at the consultation platform entrance and initiation pages, using clear and eye-catching methods, emphasizing the limitations of online consultation and providing emergency channels (e.g., hospital emergency phone numbers, psychological assistance hotlines), helping users understand and manage their expectations.

Hospitals can also, based on service operation, leverage AI's powerful data analysis capabilities and deep learning algorithms to build a hierarchical knowledge base, integrating it into the doctor-patient consultation framework to handle front-end demand docking (13, 14) (see Table 1 for details). Specifically, AI can leverage natural language processing technologies to comprehend user query intent and accurately retrieve or generate responses from a hierarchical knowledge base. For foundational information (e.g., disease definitions), AI can provide standardized knowledge-based answers. For tool-level needs (e.g., self-assessment scales), it can guide patients to utilize appropriate tools. For frequently asked questions at the consultation level, it can draw upon an authoritative response database. In this process, AI serves as an “intelligent navigator” and “initial responder,” while the knowledge base offers structured, tiered content support. Their synergy aims to enhance service efficiency and consistency, while also identifying and redirecting complex or high-risk consultations for professional human intervention.

**Table 1:** Design of a hierarchical knowledge base for new media platforms in psychiatric hospitals.

**Level**	**Content type**	**Core application Scenario**
Basic	Disease definition, symptoms, basic treatment principles.	Basic information acquisition after initial diagnosis.
Tool	Self-assessment scales, rehabilitation training guides, coping techniques.	Home management and rehabilitation support for patients.
Consultation	Authoritative Q&A library for frequent questions.	Resolving common doubts of patients and families.

Table 1 presents the hierarchical knowledge base framework designed for psychiatric hospital new media platforms, which serves as the core reference for the AI-assisted system in content matching and response generation.

Simultaneously, set up a risk word recognition and early warning mechanism, automatically prompting for manual intervention upon encountering complex or high-risk inquiries, providing “sentry” monitoring for professional intervention, and enhancing the timeliness and sensitivity of risk prevention. When necessary, hospitals can integrate user self-service query records into the Hospital Information System (HIS) to provide reference for doctors' offline consultations, forming an integrated service closed loop and improving overall operational efficiency and patient experience.

### 3.3 Breaking the dissemination efficacy bottleneck

Psychiatric hospitals can proactively connect with schools, enterprises, grassroots women's federations, and other organizations to establish long-term cooperation mechanisms. They can design “plug-and-play” science popularization packages tailored to different scenarios and needs: e.g., Freshman Psychological Adaptation Package (school entry anxiety adjustment guide paired with short videos on parent communication skills), Workplace Stress Relief Package (mindful breathing audio paired with infographics on efficient sleep), Community Elderly Cognitive Package (early dementia self-test checklist paired with instructional videos for brain exercises). These can be distributed through multi-channel promotion and precise targeting of audience push, thereby breaking through the hospital's own traffic limitations and leveraging external forces to achieve “demand-oriented” active penetration.

Hospitals can also attempt to construct an innovative three-tier ecological content system. Core content should be led by professional education teams to ensure scientific rigor and standardization. Strictly based on standardized informed consent, patients with good recovery outcomes should be encouraged to share their treatment journeys and social adaptation experiences, enhancing the authenticity and emotional resonance of the content. Collaboration with renowned experts and scholars to create branded columns focusing on the analysis of controversial topics can elevate content authority. These three elements synergize to form a content matrix of “professional foundation—patient resonance—authoritative guidance” covering the communication spectrum from basic cognition to deep needs, thereby maximizing ecological value and dissemination efficacy.

### 3.4 Innovative resource integration and long-term operation mechanisms

It is recommended that municipal-level hospitals take the lead in promoting a “regional alliance + resource sharing pool” collaboration model. A new media resource alliance can be formed, achieving intensive resource utilization and capacity co-building through a three-tier sharing pool (“basic material—technical tools—knowledge & experience” as shown in Table 2), while reducing operational costs and relying on collective wisdom to jointly formulate new media operation standards and best practices for the specialty field.

**Table 2:** Architecture of a three-tier regional alliance resource sharing pool for psychiatric hospital new media.

Level	Resource type	Core function
Basic Material	Standardized science graphics/text, universal templates, image/video material library	Avoid duplicate production, reduce basic content production costs
Technical Tool	Data analysis tool templates, operation management SOPs, training courses	Quickly enhance technical application and standardized operation capabilities of member units
Knowledge & Experience	Success case library, expert guidance documents, public opinion response strategies	Promote experience sharing and collective wisdom precipitation, establish industry best practices

Table 2 illustrates the architecture of the regional alliance's three-tier resource-sharing pool, designed to alleviate the resource constraints of individual hospitals through collective collaboration.

To tackle the talent shortage, consider adding a “Health Communication” track in professional technical title evaluations, explicitly recognizing the professional value of new media operation work, incorporating science popularization achievements into the professional technical evaluation system, and expanding the career development path for interdisciplinary talent, fundamentally providing institutional guarantee characterized by “dignity, development, and return” to attract and retain interdisciplinary talent in psychiatric specialty hospitals.

### 3.5 Building a full-cycle ethical risk control system

To address the ethical risks of new media applications in psychiatric hospitals, there is an urgent need to incorporate new media activities into the scope of ethics committee review. Guidelines for Ethical Review applicable to new media activities should be formulated and implemented based on the hospital's actual operational situation, clarifying the “three categories requiring mandatory review” principle: content planning involving stigmatizing topics related to specific diseases, research projects involving the secondary use of patient data, and technical access granting data interface permissions to third-party platforms-these three types of high-risk content must undergo review by the ethics committee, and before official content release, must be re-reviewed and approved by the ethics committee to ensure regulatory execution is in place.

The ethics committee should also establish a manual inspection mechanism, regularly sampling published content. Dynamic risk assessments should be conducted based on four aspects: “privacy security, content fairness, potential patient harm, and social trust.” Differentiated control measures should be taken according to different risk levels, establishing a “publication-monitoring-assessment-optimization” full-process risk control system to ensure the ongoing compliance and controllable risks of new media activities.

## 4 Discussion

In the new media era, the digital transformation of psychiatric specialty hospitals is an imperative. However, technology itself is a double-edged sword. Without prudent governance and effective supervision, the digitalization process cannot empower but may instead amplify the inherent drawbacks of the system. The core of the governance pathway proposed in this paper is to call for a deep recognition that new media platforms and architectures are by no means ordinary tools; they themselves are clinical intervention elements that permeate the treatment process and affect patient well-being. Therefore, how to properly manage and utilize them is a crucial starting point for psychiatric hospitals to break through the current predicament. Looking forward, we must strive to cultivate interconnected digital health and ethical literacy among clinicians, managers, and decision-makers, while simultaneously updating the policy environment to keep pace with the times, facing this unique and severe challenge through standard setting and measure implementation.

## 5 Conclusion

The application and development of new media in psychiatric hospitals must be based on ethical bottom lines and driven by management innovation. Previous evidence shows that neglecting ethics exacerbates trust crises, while technology transplantation detached from management is difficult to sustain. Only by constructing a dual-track system of “ethics-management” can the balanced development of new media empowerment and patient rights protection be achieved. This is not only the practice of the “patient-centered” concept but also an inevitable choice for psychiatric hospitals to establish their professional value and social responsibility in contemporary society.

## Data Availability

The original contributions presented in the study are included in the article/Supplementary Material, further inquiries can be directed to the corresponding author.
